# Supplementary material for: Analysis of Factors Affecting Neutralizing Antibody Production after COVID-19 Vaccination Using Newly Developed Rapid Point-of-Care Test
Source: Diagnostics (Basel). 2022 Aug 9;12(8):1924. doi: 10.3390/diagnostics12081924 (PMC9406444; doi:10.3390/diagnostics12081924)
Supplement: Supplementary file 1 [file diagnostics-12-01924-s001.zip › diagnostics-1841885-supplementary.pdf]

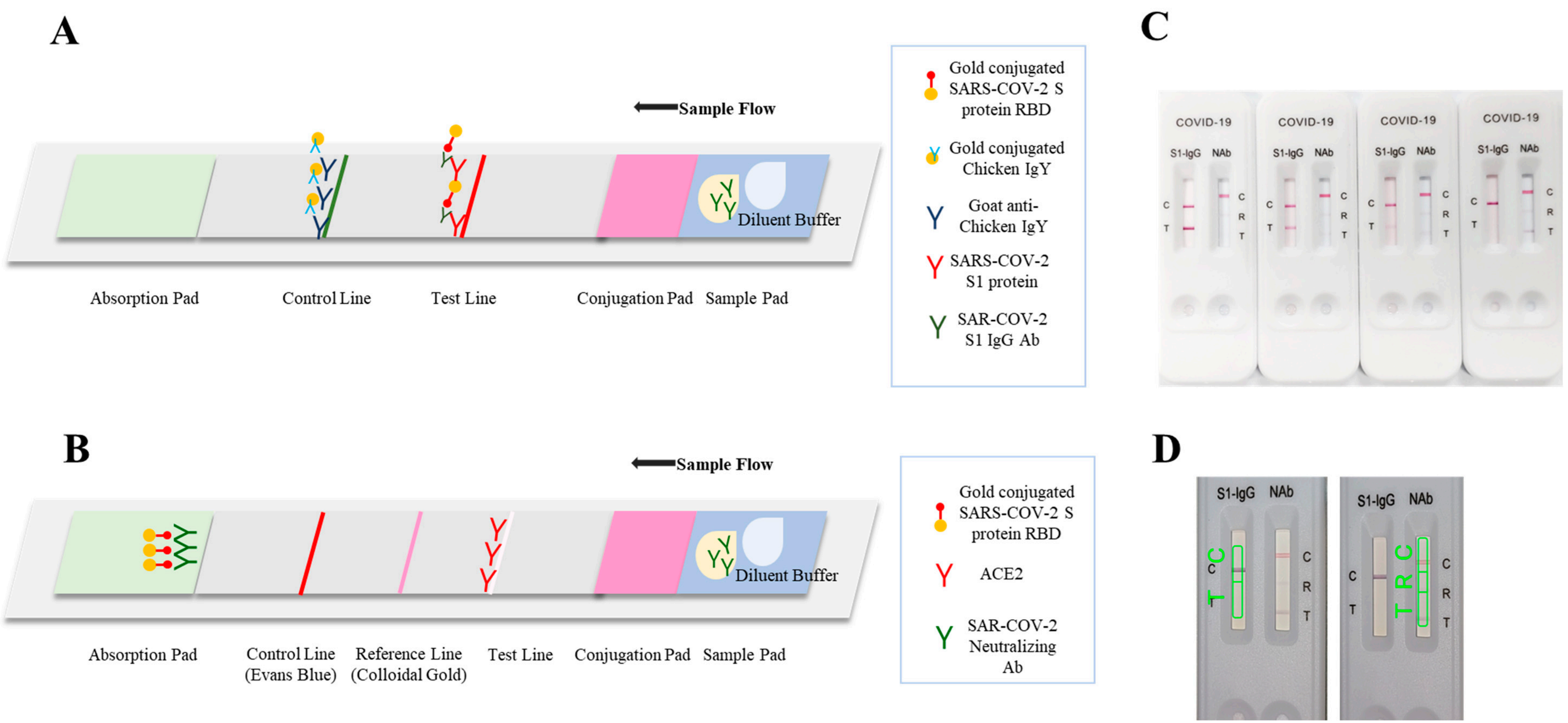

A
Sample Flow
Diluent Buffer
Absorption Pad
Control Line
Test Line
Conjugation Pad
Sample Pad
Gold conjugated SARS-COV-2 S protein RBD
Gold conjugated Chicken IgY
Goat anti-Chicken IgY
SARS-COV-2 S1 protein
SAR-COV-2 S1 IgG Ab
B
Sample Flow
Diluent Buffer
Absorption Pad
Control Line (Evans Blue)
Reference Line (Colloidal Gold)
Test Line
Conjugation Pad
Sample Pad
Gold conjugated SARS-COV-2 S protein RBD
ACE2
SAR-COV-2 Neutralizing Ab
C
COVID-19
S1-IgG
NAb
D
S1-IgG
NAb

Figure S1. RapiSure™(EDGC^{TM}) COVID-19 S1 RBD IgG/Neutralizing Ab Test. (**A**) Schematic diagram of S1 RBD IgG, (**B**) Schematic diagram of S1 RBD neutralizing antibody. The RapiSure^{TM} (EDGC^{TM}) COVID-19 S1 RBD IgG/Neutralizing Ab Test is based on the principle of lateral flow colloidal gold immunoassay for the detection of S1 RBD antibodyS1 RBD antibody and neutralizing antibody to SARS-CoV-2 in whole blood, serum or plasma. In the case of the S1 IgG test, if the sample contains S1 RBD antibody, it forms a complex with the gold-labeled antigen (RBD of the S1 spike antigen). The complex moves forward under the action of chromatography and reacts with the coated antibody (Mouse anti-human IgG monoclonal antibody) at the T band and develops color, indicating a positive result. If the sample does not contain the S1 RBD antibody, no complex will be formed at the T band and no colored band will be shown, indicating a negative result. In the case of Neutralizing Ab test, the membrane is pre-coated with Angiotensin I Converting Enzyme 2 (ACE2) on the test line region of the strip. During testing, the blood specimen reacts with S-RBD conjugated colloid gold. The mixture migrates upward on the membrane chromatographically by capillary action to react with ACE2 on the membrane and generate a colored line. The presence of this colored line indicates a negative result, while its absence indicates a positive result compare to R line. To serve as a procedural control, a colored line will always change from blue to red in the control line region, indicating that the proper specimen volume has been added and membrane wicking has occurred. (**C**) Representative patients' serum with a strong positive, positive, weak positive and negative image from left to right. (**D**) representative image of a smartphone-based image analysis SmartVision software. In the case of the S1 RBD IgG antibody, the test line within the "T" green box and the control line within the "C" green box. In the case of neutralizing antibody, the test line is within the "T" green box and the reference line is within the "R" green box. Then, the pixel intensities of the lines were automatically analyzed using the application and marked positive or negative.

Table S1. Clinical study of RapiSure™ (EDGC™) COVID-19 S1 RBD IgG/Neutralizing Ab Test

[S1 RBD- IgG Antibody]

		RT-PCR		Total
		Positive	Negative	
RapiSure™ (EDGC™) COVID-19 S1 RBD IGG/Neutralizing Ab Test	Positive	90	4	94
	Negative	3	167	170
Total		93	171	264

- Sensitivity: 96.77% (95% CI: 90.94%–98.90%)
- Specificity: 97.66% (95% CI: 94.14%–99.09%)

[Neutralizing Antibody]

		Plaque reduction neutralization Test ($PRNT_{90}$)		Total
		Positive	Negative	
RapiSure™ (EDGC™) COVID-19 S1 RBD IgG/Neutralizing Ab Test	Positive	83	0	83
	Negative	7	174	181
Total		90	174	264

- Sensitivity: 92.22% (95% CI: 84.81%–96.18%)
- Specificity: 100.00% (95% CI: 97.84%–100.00%)

Table S2. Agreement between naked eye and measurements application interpretation results of RapiSure™ (EDGC^{TM}) COVID-19 S1 RBD IgG/Neutralizing Ab Test

[S1 RBD IgG Antibody]

RapiSure™ (EDGC^{TM}) COVID-19 S1 RBD IgG/Neutralizing Ab Test		Application		Total
		Positive	Negative	
Naked eye	Positive	635	0	635
	Negative	1	100	101
Total		636	100	736

- Positive percent agreement (PPA) (%) = 99.84 (CI: 99.11~99.97)
- Negative percent agreement (NPA) (%) = 100.00 (CI: 96.30~100.00)
- Total percent agreement (TPA) (%) = 99.86 (CI: 99.23~99.98)
- Cohen′s kappa(κ) = 0.992

[Neutralizing Antibody]

RapiSure™ (EDGC^{TM}) COVID-19 S1 RBD IgG/Neutralizing Ab Test		Application		Total
		Positive	Negative	
Naked eye	Positive	483	2	485
	Negative	3	248	251
Total		486	250	736

- Positive percent agreement (PPA) (%) = 99.38 (CI: 98.20~99.79)
- Negative percent agreement (NPA) (%) = 99.20 (CI: 97.13~99.78)
- Total percent agreement (TPA) (%) = 99.32 (CI: 98.42~99.71)
- Cohen′s kappa(κ) = 0.992

Table S3. Comparison of commercial serological SARS-CoV2 Neutralizing antibody assay with RapiSure™ (EDGC^{TM}) COVID-19 S1 RBD IgG/Neutralizing Ab Test

[Neutralizing Antibody]

		ELISA		Total
		Positive	Negative	
RapiSure™ (EDGC^{TM}) COVID-19 S1 RBD IGG/Neutralizing Ab Test	Positive	471	6	477
	Negative	15	244	259
Total		486	250	736

- Positive percent agreement (PPA) (%) = 96.91 (CI: 94.97~98.12)
- Negative percent agreement (NPA) (%) = 97.60 (CI: 94.86~98.90)
- Total percent agreement (TPA) (%) = 97.15 (CI: 95.68~98.13)
- Cohen′s kappa(κ) = 0.992
